# Unveiling nonlinear effects of Digital Inclusive Finance on urban-rural integration: A threshold panel analysis of China

**DOI:** 10.1371/journal.pone.0342432

**Published:** 2026-02-20

**Authors:** Ying Song, Yuanping Cao, Zhiyi Zhuo, Feifei Yang

**Affiliations:** 1 Green Development Research Center, Wuyi University, Fujian, China; 2 School of Economics and Management, Zhejiang Ocean University, Zhejiang, China; Pusan National University College of Economics and International Trade, KOREA, REPUBLIC OF

## Abstract

This paper examines the nonlinear effects of Digital Inclusive Finance (DIF) on urban–rural integration (URI) using a provincial panel for mainland China (31 provinces, 2011–2023). We construct a multidimensional URI index and decompose DIF into coverage breadth (D1), usage depth (D2) and digitalization level (D3). Estimation proceeds with two-way fixed-effects models and Hansen-style panel threshold regressions with bootstrap inference; robustness checks include placebo tests and instrumental-variable specifications. The evidence shows that DIF’s impact on URI is regime-dependent: marginal returns are limited at low development levels but increase sharply once DIF and complementary institutional conditions cross empirically identified thresholds. Disaggregation reveals that usage depth (D2) consistently promotes integration, whereas the benefits of coverage (D1) and digitalization (D3) materialize mainly in digitally mature regimes. Traditional finance exhibits declining marginal contribution beyond its effective range, underlining the catalytic role of digital systems. We document heterogeneity across regions and show that negative baseline coefficients on openness and education reflect spatial concentration rather than intrinsic harms. The findings reconcile mixed results in prior work and imply that policy should be threshold-aware: prioritize foundational access where coverage is low, while in advanced contexts emphasize usage, platform interoperability, and regulatory safeguards to manage platform concentration and distributional risks.

## Introduction

Urban-rural integration (URI) is a core component of China’s development agenda, aiming to promote spatial equity, balanced growth, and domestic circulation. Despite decades of reform, a persistent urban–rural divide continues to constrain economic efficiency and social cohesion [1]. While income gaps have narrowed, disparities in infrastructure, public services, and capital access remain substantial [2]. Addressing these multidimensional imbalances is essential to achieving high-quality, inclusive development.

A fundamental obstacle to URI lies in the unequal distribution of financial resources. China’s financial system has long favored urban regions, resulting in systematic underinvestment in rural economies and reinforcing institutional exclusion [3]. Although reforms have improved access, capital misallocation remains a structural constraint.

In this context, Digital Inclusive Finance (DIF) has emerged as a transformative force. Powered by mobile technologies, big data, and AI platforms, DIF reduces transaction costs and information asymmetries, expanding access to credit, insurance, and payments in underserved regions [4]. Compared to traditional finance, DIF is decentralized and adaptive, offering new pathways for rural revitalization and economic inclusion.

However, research on the DIF–URI nexus remains limited. Existing studies have mainly focused on income inequality, overlooking URI as a multidimensional concept encompassing economic, social, spatial, ecological, and demographic integration. Moreover, few have explored potential nonlinearities—whether DIF exerts stronger influence once it surpasses certain development thresholds—or decomposed DIF into its core subdimensions (coverage, usage, and digitalization) to uncover distinct mechanisms.

This paper advances the literature in four ways. First, it shifts from income-based measures to a multidimensional URI index, allowing a holistic assessment of integration outcomes. Second, this paper contributes to the literature by explicitly linking the digital-finance–integration nexus to threshold theory and regime dependence. Building on Hansen [5], we posit that the developmental returns to Digital Inclusive Finance (DIF) are nonlinear: in early stages, limited infrastructure and low digital literacy constrain the effective use of DIF; beyond empirically identifiable thresholds, network effects, interoperability, and institutional complementarities amplify marginal returns. Empirically estimating these thresholds therefore yields direct policy-relevant guidance on when and where digital-finance interventions are most likely to produce transformative integration outcomes. Third, the study disaggregates DIF into its subdimensions to examine their differential contributions to URI. Fourth, it explores whether digital and traditional finance act as complements or substitutes in fostering integration.

Using balanced provincial panel data from 2011–2023, we employ two-way fixed effects, panel threshold models, and instrumental-variable strategies. The results reveal pronounced nonlinear returns to DIF, strong spatial heterogeneity, and context-specific financial interactions. These findings not only enrich the understanding of China’s integration experience but also contribute to the global inclusive-finance literature, in which many developing economies face similar challenges of unequal financial access, uneven digital readiness, and regionally heterogeneous development. The framework and empirical approach adopted here provide a replicable analytical tool for assessing digital-finance-led integration processes beyond China.

The remainder of this paper is organized as follows. Section “Literature review” reviews the literature on financial development, inclusive finance, DIF, and URI, identifies research gaps, and outlines the research questions. Section “Theoretical framework and hypotheses” develops the theoretical framework, explaining the mechanisms through which DIF influences URI via coverage, usage, and digitalization pathways, and proposes the corresponding hypotheses. Section “Research methodology” presents the data, variable definitions, and econometric methods, including the entropy-based construction of the URI index, the core explanatory variables (Peking University’s DIF Index and its subdimensions), and the specification of the threshold and fixed-effects models. Section “Results” report the empirical results, including baseline regressions, subdimension analyses, and regional heterogeneity tests. Section “Robustness checks” conduct robustness checks using alternative measures (a broader digital economy index), sample screening, placebo tests, and instrumental-variable approaches, with additional diagnostics for multicollinearity. Section “Threshold effects test” examines threshold effects and interprets their institutional implications across development stages. “Discussion and conclusion” section discuss theoretical contributions and policy implications, followed by the conclusion summarizing key findings and limitations. The Appendix details the indicator system, entropy computations, and data sources.

## Literature review

### Theoretical evolution of financial development and inclusive finance

Financial development has long been recognized as a driver of economic transformation, guided by classical theories such as financial intermediation [6] and the financial repression hypothesis [7]. In developing economies, financial deepening promotes growth and reduces poverty by improving capital allocation and lowering transaction costs, but its redistributive effects are conditional on institutional quality, governance and human capital [8,9]. Empirical studies indicate that without institutional safeguards, financial expansion may favor high-income urban groups, exacerbating inequality [9]. Nonlinear effects have also been documented: initial stages of financial deepening may narrow income gaps, whereas mature markets may reinforce disparities [10]. These findings motivate the emergence of inclusive finance, which explicitly addresses structural exclusion within conventional financial systems. Evidence shows that inclusive finance fosters poverty alleviation and regional equity, though spatial heterogeneity persists [11].

### The transition from inclusive finance to DIF

The rise of ICT and fintech has transformed financial inclusion. Digital Inclusive Finance (DIF), encompassing mobile payments, digital credit, insurance, and data-driven risk assessment, reduces service barriers and broadens access for rural populations and microenterprises [12–14]. Comparative research highlights contrasting adoption patterns: advanced economies emphasize product innovation and regulatory adaptation, while developing economies rely on mobile platforms and agent-banking to overcome infrastructure deficits [15,16]. In China, DIF supports rural revitalization, agricultural modernization, and employment growth, with stronger effects in less-developed western provinces and diminishing returns in mature eastern regions [17–19]. Evidence from India confirms that digital financial inclusion reduces income inequality [20]. Yet potential downsides exist: unequal digital literacy and platform concentration may reinforce exclusion [21–24].

Despite this growing body of work, several gaps remain. First, most studies emphasize income distribution rather than broader dimensions of urban–rural integration (URI), including social-service equity, spatial mobility, and ecological sustainability. Second, the literature rarely considers nonlinear dynamics—for example, whether DIF only becomes effective beyond critical thresholds [25,26]. Third, regional heterogeneity is underexplored, particularly how local financial structures, policy environments, and demographic conditions shape DIF’s impact.

### From DIF to URI: Theoretical linkages and research gaps

While most DIF research focuses on income or poverty outcomes, URI is multidimensional, encompassing economic convergence, equitable access to services, spatial connectivity, ecological sustainability, and demographic mobility [13,27]. Understanding DIF’s impact requires analyzing its transmission channels—credit expansion, digital payments, insurance, and platform services—within local institutional contexts. Nonlinear and threshold effects are increasingly recognized: DIF may only significantly influence URI beyond critical levels of technology diffusion or institutional readiness [25,26]. To address these gaps, this study decomposes DIF into coverage breadth, usage depth, and digitization level, applying a panel threshold regression model to examine regime-dependent effects across five URI dimensions using provincial data for China (2011–2023). This approach informs both China’s policy agenda and comparative analyses of inclusive digital transformation in developing economies.

## Theoretical framework and hypotheses

### Conceptual framework

URI involves coordinating economic structures, social services, population mobility, and spatial development across urban and rural areas. Achieving URI requires overcoming barriers in capital flows, institutional access, and infrastructure.

DIF offers opportunities to reshape urban–rural linkages by reducing financial frictions and expanding participation in formal financial systems [28,29]. Drawing on the inclusive innovation framework [30], DIF may enhance URI through two channels: (1) economic empowerment—facilitating credit access, lowering transaction costs, supporting rural entrepreneurship, and stimulating industrial upgrading; (2) social transformation—expanding digital platforms, improving e-commerce and tele-services, and integrating digitally mediated public infrastructure. DIF may also generate positive externalities that reinforce rural governance and environmental quality, indirectly supporting URI [17,18].

**Hypothesis 1:** Higher levels of DIF are associated with enhanced URI by improving financial access and enabling broader economic and institutional participation in rural areas.

### Threshold effects and regional heterogeneity

The impact of DIF on URI is unlikely to be linear or uniform. In regions with low internet penetration or limited digital literacy, DIF’s effects may be constrained; as digital ecosystems mature, benefits may accelerate, suggesting threshold effects [27,29,31]. Prior studies emphasize that digital technologies often produce significant impacts only after surpassing critical levels of infrastructure and institutional capacity [25,26].

China’s regional diversity in institutional quality, policy support, and human capital further implies spatial heterogeneity. For example, rural households may benefit more from digital financial access than urban counterparts [32], highlighting differentiated inclusion dynamics.

**Hypothesis 2:** The relationship between DIF and URI exhibits threshold characteristics. The early stages of DIF development have limited effects, while beyond a certain threshold, impacts strengthen substantially.

**Fig 1:**
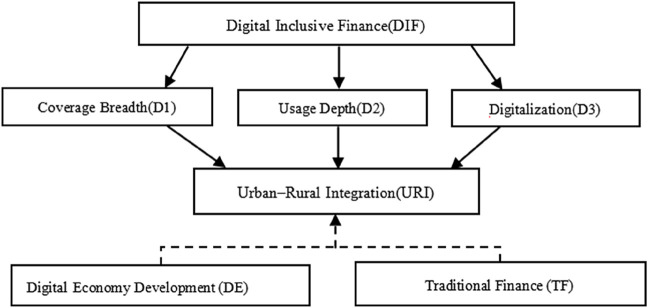
Conceptual framework of DIF and URI. Note: DIF includes three sub-indices (D1, D2, D3). Threshold effects are modeled for DIF, sub-indices, and traditional financial development (TF).

Fig 1 illustrates the proposed causal pathways and nonlinear dynamics between DIF and URI.

## Research methodology

### Variable selection

#### Dependent variable: URI.

To measure URI at the provincial-level, this study constructs a composite index grounded in the core connotations of integration—economic linkage, social equalization, spatial connectivity, ecological coordination, and demographic restructuring. This multidimensional framework aligns with the objectives of China’s New Urbanization Plan (2014–2020) and builds on established empirical studies [33–35], ensuring both conceptual rigor and policy relevance.

The selection of indicators follows three principles. First, indicators must reflect the essential mechanisms of URI. Economic disparity, public-service provision, spatial mobility, ecological sustainability, and demographic transitions represent the fundamental domains through which integration occurs. Second, indicators are chosen for their ability to reveal meaningful temporal and cross-provincial differences, allowing the entropy method to extract useful information and avoid redundancy. Third, all variables are sourced from standardized and publicly available statistical yearbooks to ensure reliability and comparability.

#### Economic dimension.

Income, consumption, and industrial-structure gaps directly reflect the degree of factor reallocation across urban and rural sectors. Reductions in these disparities indicate convergence in living standards and production patterns—core aspects of integration.

#### Social dimension.

Differences in education, healthcare, cultural services, and social-security coverage capture institutional equalization. URI presupposes that residents across regions can access comparable welfare and public-service systems.

#### Spatial dimension.

Indicators related to transportation networks, information accessibility, and urban–rural mobility reflect the reduction of physical and informational frictions. Spatial connectivity is indispensable for labor migration, goods circulation, and service delivery.

#### Ecological dimension.

Measures of energy efficiency, pollution intensity, environmental spending, and ecological resources assess whether integration is compatible with green development. URI, as a long-term development process, requires ecological constraints and governance capacities to be incorporated.

#### Demographic dimension.

Urbanization rates, employment structure, and population mobility reflect the reconfiguration of human–land relationships. These transitions are the most visible manifestations of integration depth.

Weights are assigned using the entropy method, which captures the relative information content of each indicator in a data-driven manner. Compared with subjective or equal-weight approaches, entropy weighting improves transparency and sensitivity to regional heterogeneity. The final URI index is computed as the weighted sum of standardized indicators, providing a comprehensive measure of integration across provinces. Data sources, full indicator descriptions and the entropy method are provided in S1 File, S2 Appendix (S2 Table 1–2) and S3 Appendix.

#### Independent variable: DIF.

The core explanatory variable is the DIF Index, compiled by the Digital Finance Research Center at Peking University and available for all provinces from 2011 to 2023. The index comprises 31 sub-indicators grouped into three core dimensions: coverage breadth (D1), usage depth (D2), and digitalization level (D3). Collectively, these dimensions reflect the accessibility, penetration, and technological sophistication of digital financial services across regions.

The DIF index has been widely used in empirical research exploring the intersections of financial inclusion, digitalization, and rural development in China. It is considered a robust indicator of the digital financial ecosystem’s maturity and serves as the focal variable in examining its effect on URI.

Since the original values of DIF range from approximately 0 to 300, we follow the convention adopted in recent empirical studies [36] by scaling the index through division by 100. This standardization brings the values into a more interpretable range (0–3), facilitating coefficient comparison, improving numerical stability in regressions, and maintaining consistency across all sub-indicators. Importantly, this transformation does not affect the direction or statistical significance of the results but facilitates easier interpretation of the regression coefficients by harmonizing the scale with other variables.

#### Control variables.

Macro-level controls include: Fixed Capital Investment (FCI: total fixed asset investment/GDP), Fiscal Support for Agriculture (GOV: agricultural fiscal expenditure/output), Openness (OPEN: trade/GDP), Education Development (EDU: education expenditure/GDP), and Mobile Phone Penetration (MPP: subscriptions per 100 people).

While these control variables enhance the robustness of the model, we acknowledge potential multicollinearity—particularly between EDU and certain components of the URI index (e.g., public service delivery). We address this issue in Section Results by reporting variance inflation factors (VIFs) and conducting additional robustness checks.

#### Threshold and benchmark variables.

To capture nonlinear dynamics, the three DIF sub-indices(DI-D3) serve as threshold variables in the panel threshold regression framework [5]. A Traditional Financial Development (TF) index is constructed using principal component analysis (PCA) on the number of commercial bank branches and employees, providing a benchmark for comparison with DIF.

The TF index is based on two indicators: (1) the number of commercial bank branches and (2) the number of employees in commercial banking institutions. After applying natural logarithmic transformation to correct for skewness, we use PCA to extract the first principal component, which captures the dominant pattern in traditional financial development across provinces. This provides a basis for comparison with the digital financial ecosystem. Data for the TF index are obtained from the China Financial Statistical Yearbook and the China Banking and Insurance Statistical Bulletin.

### Model specification

To estimate the impact of DIF on URI, we begin with a two-way fixed-effects specification that controls for time-invariant provincial characteristics and common temporal shocks. The baseline regression equation is formulated as follows:

$${URI}_{it}=\alpha_{i}+\gamma_{t}+\beta {DIF}_{it}+\delta^{\prime }X_{it}+\varepsilon_{it}$$ (1)

where $\alpha_{i}$ and $\gamma_{t}$ denote province and year fixed effects, *X*_*it*_ is the vector of control variables (FCI, GOV, OPEN, EDU, MPP), and $\varepsilon_{it}$ is the idiosyncratic error term. A fixed-effects estimator is preferred over random effects because regional governance quality, infrastructure, and economic structure are likely correlated with financial development, making the strict exogeneity assumption implausible.

While Eq (1) identifies the average association between DIF and URI, theoretical considerations and prior digital-finance research suggest that returns to digitalization may be nonlinear. Early-stage digital ecosystems suffer from low interoperability and limited digital literacy, whereas mature ecosystems generate stronger network and institutional complementarities. To capture this regime dependence, we apply Hansen’s (1999) panel threshold model:

$${URI}_{it}=\left\{ \begin{array}{cc} \alpha_{i}+\gamma_{t}+\beta_{1}{DIF}_{it}+\delta^{\prime }X_{it}+\varepsilon_{it}, & q_{it}\leq \tau_{1}\\ \alpha_{i}+\gamma_{t}+\beta_{2}{DIF}_{it}+\delta^{\prime }X_{it}+\varepsilon_{it}, & \tau_{1}<q_{it}\leq \tau_{2}\\ \alpha_{i}+\gamma_{t}+\beta_{3}{DIF}_{it}+\delta^{\prime }X_{it}+\varepsilon_{it}, & q_{it}>\tau_{2} \end{array}\right.$$ (2)

where *q*_*it*_ is the threshold variable (DIF or one of its sub-indices), and $\tau_{1}$, $\tau_{2}$ are estimated endogenously. Inference relies on bootstrap methods with 1,000 replications following Hansen (1999). MPP was excluded from threshold estimations because its strong correlation with DIF led to unstable regime classification in bootstrap iterations; excluding it does not materially affect the results.

By integrating fixed-effects and threshold approaches, our empirical strategy identifies not only the average effect of DIF on URI but also how this effect changes across stages of digital-financial development, enabling a more precise test of the nonlinear “digital maturity” hypothesis.

## Results

This study employs an unbalanced panel of 31 provincial-level regions in mainland China for the period 2011–2023. Hong Kong, Macao, and Taiwan are excluded due to institutional and statistical incompatibility, ensuring cross-provincial comparability and internal validity. All variables are sourced from authoritative national databases, including the *China Statistical Yearbook*, provincial statistical yearbooks, the *China Financial Statistical Yearbook*, the *China Education Statistical Yearbook*, and the *China Banking and Insurance Statistical Bulletin*. The DIF index and its three sub-indices (D1–D3) are drawn from the Peking University Digital Finance Research Center.

The sample captures major structural transformations and exogenous shocks—most notably the COVID-19 pandemic—which reshaped labor mobility, digital consumption, and fiscal responses. To absorb such macro-level fluctuations, all regressions include year-fixed effects. Variable selection is guided by theoretical mechanisms linking DIF to URI: economic empowerment, transaction-cost reduction, institutional transformation, and public-service equalization.

### Descriptive statistics and multicollinearity diagnostics

Table 1 reports descriptive statistics for key variables. The URI index exhibits substantial cross-provincial variation (mean = 0.202; SD = 0.076), reflecting heterogeneous integration levels. The DIF index and its sub-indices also show broad dispersion, consistent with uneven digital development across regions. MPP is retained in its original unit to capture infrastructure capacity without information loss.

**Table 1:** Descriptive statistics of key variables.

Variable Name	Symbol	Mean	SD	Min	Max	Obs
**Dependent Variable**						
Urban-Rural Integration Development Level	URI	0.2017	0.0755	0.0727	0.6396	403
**Independent Variable**						
Digital Inclusive Finance Development Index	DIF	2.5445	1.1133	0.1622	4.7383	403
**Control Variables**						
Fiscal Support Level	GOV	5.5873	2.8237	0.8842	13.992	403
Level of Openness to the Outside World	OPEN	0.2583	0.2795	0.0077	1.538	403
Education Development Level	EDU	0.0425	0.0234	0.0211	0.1733	403
Fixed Capital Investment Level	FCI	0.7809	0.2379	0.2121	1.6196	403
Mobile Phone Penetration	MPP	104.05	25.3591	52.04	189.46	403
**Threshold Variables**						
Breadth of Coverage (DIF)	D1	2.3925	1.1613	0.0196	4.6654	403
Depth of Usage (DIF)	D2	2.4555	1.0957	0.0676	5.1069	403
Degree of Digitalization (DIF)	D3	3.2101	1.1779	0.0758	4.7689	403
Traditional Financial Development Level	TF	$-2.62\times 10^{-17}$	1.4043	–4.249	2.2792	403

Multicollinearity is assessed using VIFs; results are shown in Table 2. Most control variables have VIFs below 5. However, the DIF sub-indices (D1–D3) exhibit higher VIFs due to their shared origin in a composite index. To avoid mechanical multicollinearity, we exclude the composite DIF when estimating models with sub-indices and vice versa. Robustness checks excluding EDU or rescaling D1–D3 confirm that coefficients for DIF and D2 remain stable, indicating that collinearity does not drive our main findings.

**Table 2:** Variance inflation factor (VIF) results.

Variable	VIF	1/VIF
Fiscal Support Level (GOV)	3.77	0.2652
Level of Openness (OPEN)	3.18	0.3143
Education Development (EDU)	3.07	0.3260
Fixed Capital Investment (FCI)	2.17	0.4606
Mobile Phone Penetration (MPP)	5.00	0.1997
Breadth of Coverage (D1)	21.95	0.0456
Depth of Usage (D2)	11.99	0.0834
Degree of Digitalization (D3)	6.35	0.1576
Traditional Finance (TF)	6.21	0.1609

Notes: The table reports the VIF values to assess multicollinearity among explanatory variables in the baseline model. The variable DIF was excluded from the model due to perfect collinearity with its sub-indices (D1-D3). Consequently, it does not appear in the VIF statistics. D1, D2, and D3 exhibit inherently high correlation by construction. VIF values exceeding 10 are generally considered indicative of high multicollinearity.

### Baseline regression results

Table 3 reports the baseline regression results examining the association between DIF and URI. Model (1) includes only year and province fixed effects. The estimated coefficient for DIF is 0.1302 (*p* = 0.022), indicating a statistically significant and positive association with URI.

**Table 3:** Benchmark regression results.

Variables	Model (1)	Model (2)
DIF	0.1302^**^	0.0896^***^
	(0.0537)	(0.02799)
FCI	–	0.001
		(0.0085)
GOV	–	–0.00186
		(0.0019)
OPEN	–	–1.1158^***^
		(0.0350)
EDU	–	–0.3897^**^
		(0.1923)
MPP	–	0.0005
		(0.0005)
Constant	0.1032^***^	0.1399^**^
	(0.0237)	(0.0430)
Year fixed effect	Yes	Yes
Province fixed effect	Yes	Yes
Observations	403	403
Within-*R*^2^	0.7416	0.8027

Notes: ^***^p < 0.01, ^**^p < 0.05,  *p* < 0.1. Robust standard errors clustered at province level in parentheses. Dashes (–) indicate variables not included in the model.

Model (2) incorporates additional control variables—FCI, GOV, OPEN, EDU, and MPP. The DIF coefficient remains significant (β=0.0896, *p* = 0.003), underscoring its robust impact. The within-*R*^2^ increases to 0.803, suggesting improved explanatory power.

Interestingly, the coefficients for OPEN and EDU are negative and statistically significant, suggesting potential structural asymmetries in how openness and education affect integration. This pattern warrants further exploration, which we address through decomposition and interaction analyses in “Decomposition Tests for OPEN and EDU”. Overall, the results confirm a robust and positive association between DIF and URI, though we refrain from asserting strict causality due to potential endogeneity concerns.

### Sub-dimensional analysis of DIF

To explore underlying mechanisms, we decompose DIF into its three sub-indices—D1 (coverage breadth), D2 (usage depth), and D3 (digitalization degree)—and re-estimate the regression models. Table 4 presents the findings.

**Table 4:** Results of DIF sub-indices.

	DIF	D1	D2	D3
**Panel A. National Sample (n=403)**				
Coefficient	0.0896^***^	0.0233	0.0532^***^	0.0293^***^
	(0.0279)	(0.0199)	(0.0162)	(0.0096)
Controls	Yes	Yes	Yes	Yes
FE (Year & Province)	Yes	Yes	Yes	Yes
**Panel B. Eastern Region (n=143)**				
Coefficient	0.0189*	0.0177	0.0172^**^	0.0071
	(0.0090)	(0.0088)	(0.0072)	(0.0051)
Controls	Yes	Yes	Yes	Yes
FE (Year & Province)	Yes	Yes	Yes	Yes
**Panel C. Central Region (n=104)**				
Coefficient	0.0181^***^	0.0184^***^	0.0012	0.0076^***^
	(0.0021)	(0.0028)	(0.0021)	(0.0011)
Controls	Yes	Yes	Yes	Yes
FE (Year & Province)	Yes	Yes	Yes	Yes
**Panel D. Western Region (n=156)**				
Coefficient	0.1440^***^	0.0147^***^	0.0110^***^	0.0059^***^
	(0.0018)	(0.0016)	(0.0021)	(0.0012)
Controls	Yes	Yes	Yes	Yes
FE (Year & Province)	Yes	Yes	Yes	Yes

Notes: ^***^p < 0.01, ^**^p < 0.05,  *p* < 0.1. Robust standard errors clustered at province level in parentheses. Each column reports estimates from a separate regression model using the indicated sub-indices of DIF.

Only D2 and D3 are statistically significant and positively associated with URI, while D1 is not. This suggests that mere access to digital financial tools is insufficient; what matters more is active engagement and technological sophistication. These findings align with existing literature emphasizing usage over access in achieving meaningful financial inclusion outcomes.

D2 is positive and significant at the 1% level (*p* = 0.003), highlighting the importance of transaction frequency and sustained financial behavior. D3 is also significant (*p* = 0.005), pointing to the critical role of technological infrastructure and digital service quality. In contrast, D1 is not statistically significant (*p* = 0.252), indicating that expanded access without corresponding use may not contribute to URI.

These results reinforce the need for policies that promote not just access to, but also meaningful usage of, digital financial tools—especially in rural and underserved areas.

As shown in Table 2, the variance inflation factors (VIFs) for the sub-dimensions of DIF—particularly D1 and D2—exceed the conventional threshold of 10. This reflects their intrinsic interdependence, as these components originate from the same composite index and tend to evolve jointly in practice: wider coverage typically coincides with deeper usage and higher digitalization. To ensure robustness and interpretive reliability, we employ three complementary strategies.

(1) *Stepwise regression comparison.* The theoretical analysis does not rely solely on coefficient magnitudes within a single specification. Instead, we estimate stepwise regressions by introducing each sub-dimension separately and jointly to compare their relative effects. This approach reveals that D2 consistently exerts a positive and statistically significant influence on URI across all model specifications, while the signs and interpretations of D1 and D3 remain stable.

(2) *Principal component adjustment.* To control for shared variance among sub-dimensions, we apply principal component analysis (PCA) to extract their common component and re-estimate the baseline model. The resulting PCA-based index remains positive and significant at the 1% level (β=0.0477, *p* = 0.002). This consistency indicates that the observed effects of DIF on URI are not artifacts of multicollinearity.

(3) *Ridge regression validation.* Finally, we implement ridge regression using the optimal penalty parameter (α=79.25) determined via cross-validation. The ridge estimates preserve the same sign pattern as the OLS coefficients, with only minor magnitude adjustments (see S4 Table). This confirms that the positive association between DIF and URI remains stable even when multicollinearity is explicitly penalized.

Across all robustness checks, three conclusions emerge: (i) the positive effect of D2 is stable and significant in every model; (ii) when multiple sub-indices are included simultaneously, statistical significance declines slightly, but coefficient signs and economic interpretations remain unchanged; and (iii) PCA- and ridge-based estimations yield nearly identical substantive results.

Hence, while multicollinearity among sub-dimensions is acknowledged, it does not distort the estimated relationships or alter substantive conclusions. Retaining the sub-dimensional structure is theoretically justified, as it reveals the differentiated transmission mechanisms through which digital inclusive finance promotes URI.

### Regional heterogeneity analysis

To capture the contextual diversity of DIF effects on URI, this section extends the baseline regressions to explore heterogeneity across multiple structural dimensions. Given space constraints, we focus on the two most theoretically salient sources of variation—regional differences and digital infrastructure levels—while briefly summarizing additional robustness tests. Detailed results are presented in Table 4 and (S5 Table 1–4).

1. **Regional Heterogeneity**
  Spatial differences in China remain the most fundamental dimension of economic and institutional variation. Subsample regressions for the eastern, central, and western regions reveal clear gradients in the effectiveness of DIF (see Table 4).
  In the eastern region, where digital and financial infrastructure is already mature, DIF’s overall effect on URI is positive but only marginally significant (β=0.0189, *p* = 0.062). This suggests diminishing marginal returns; incremental digital-finance expansion yields limited additional gains once access saturation has been achieved.
  In the central region, coefficients for all sub-dimensions are positive and significant, reflecting a “sweet spot” where digital finance interacts with improving infrastructure and growing rural demand to produce balanced integrative outcomes.
  In contrast, the western region shows the strongest effects (β=0.144, *p* < 0.001) across all dimensions, indicating that DIF serves a substitutive function—compensating for physical and institutional deficits by enabling access, connectivity, and entrepreneurial opportunity.
  Overall, these results highlight a spatially stratified pattern. DIF yields the highest marginal benefits in transitional or lagging regions where traditional financial penetration remains low and digital technologies can leapfrog structural bottlenecks.
2. **Heterogeneity by ICT Infrastructure**
  To further test how technological readiness shapes the inclusivity of digital finance, provinces are divided into high-ICT and low-ICT groups based on internet penetration. Results (S5 Table2) indicate that DIF’s positive impact on URI is robust across both groups but substantially stronger in high-ICT provinces (β=0.112, *p* < 0.01) than in low-ICT ones (β=0.070, *p* < 0.05). The significant interaction term between DIF and ICT underscores a complementarity mechanism: advanced digital infrastructure amplifies the effectiveness of financial digitalization by improving transaction efficiency, lowering service costs, and facilitating real-time data verification.
  Among sub-indices, D2 and D3 display the highest sensitivity to ICT conditions, confirming that the transformative power of DIF depends not only on policy support but also on the technological ecosystem in which it operates.
3. **Additional Heterogeneity Tests**
  Three supplementary robustness checks further corroborate these findings:
  - Grouping provinces by economic development stage (per capita GDP) shows that DIF’s marginal effect strengthens with higher development levels, yet the “usage depth” channel remains significant even in low-income provinces.
  - Heterogeneity by industrial structure (service-sector share) suggests stronger DIF effects in service-oriented economies, where small-scale and digital-intensive activities dominate.
  - Analysis by fiscal support intensity reveals a positive interaction between public expenditure and DIF, implying that well-targeted fiscal programs can crowd in digital-finance participation rather than substitute for it.

Collectively, these tests confirm that DIF’s integrative impact is conditioned by both structural maturity and institutional coordination. Across all dimensions, two consistent findings emerge: (i) D2 remains the most stable and significant driver of URI, and (ii) DIF’s marginal returns rise with improvements in infrastructure, development level, and public support. These results demonstrate that digital finance is most transformative when embedded in an enabling technological and institutional environment.

### Decomposition tests for OPEN and EDU

To further clarify the unexpected negative or insignificant coefficients on OPEN and EDU in the baseline results, we conduct decomposition and interaction analyses to explore whether these effects are driven by urban concentration rather than true “disbenefits” of openness or education.

Specifically, we proxy urban- and rural-oriented education by interacting total education investment with the provincial urbanization rate (edu_total × Urban_rate) and its complement (edu_total × (1 – Urban_rate)). We also construct an openness–urbanization interaction term (OPEN × Urban_rate) and an export-intensity variable (export_share = exports/GDP) to capture differences in the spatial concentration of trade activities.

The results are reported in S6 Table. Across all specifications, the main findings are consistent and intuitive:

- The coefficients on edu_urban_proxy and edu_x_urban are small and statistically insignificant, whereas the rural-oriented component (edu_rural_proxy) remains weakly negative. This pattern suggests that the overall negative EDU effect in the baseline regression stems largely from the urban bias of education investment and the associated outflow of human capital from rural areas. When accounting for urbanization heterogeneity, the education effect on URI becomes economically small and no longer significant, indicating that the negative sign does not reflect an inherently harmful role of education but rather resource diversion toward urban cores.
- Regarding openness, the coefficient on open_x_urban is strongly negative and highly significant (β=−0.443, *p* < 0.01), whereas the baseline OPEN effect becomes positive when this interaction is included (β=0.231, *p* < 0.01). This confirms that the negative impact of openness is concentrated in provinces where trade and investment benefits accrue primarily to urbanized regions. Similarly, the export_share variable itself shows no robust association with URI once spatial concentration is controlled for, implying that trade intensity per se is not harmful, but its urban-centric distribution can temporarily inhibit province-level integration.
- The grouped regressions reinforce this mechanism: for provinces below the median urbanization or export concentration, the coefficients on OPEN and EDU are close to zero or slightly positive, whereas in highly urbanized or trade-concentrated provinces, the OPEN effect turns sharply negative. Taken together, these results support the interpretation that the apparent negative signs of OPEN and EDU are artifacts of urban concentration and center–periphery dynamics, not genuine detrimental effects on integration.

These findings enrich our understanding of the role of structural asymmetries in digital and economic development. They underscore that openness and education can promote integration only when accompanied by mechanisms that channel their benefits beyond urban cores, such as rural education enhancement, balanced fiscal transfers, and inclusive trade-link infrastructure.

## Robustness checks

### Replacing DIF with digital economy development

To assess robustness, we re-estimate the benchmark model by replacing the DIF index with a broader indicator of Digital Economy development (DE). Conceptually, DE and DIF are related but not interchangeable. The digital economy encompasses economy-wide digitalization—including ICT infrastructure, industrial digital transformation, e-commerce, and data-driven business models—whereas DIF refers specifically to the digitalization and diffusion of financial services. This hierarchical distinction is well established in the literature, which characterizes digital finance as a component within the broader digital economy system [37,38].

Thus, substituting DIF with DE constitutes an upper-category robustness test rather than a direct conceptual replacement. If both indicators yield similar signs and significance, this suggests that general digitalization and financial digitalization jointly promote URI; divergence would imply that DIF operates through mechanisms not captured by the broader DE construct. To avoid misinterpretation, we explicitly report DE as an alternative variable and interpret its coefficient as reflecting broad digitalization effects instead of financial-channel mechanisms.

Empirical results (Table 5) show that DE remains positively associated with URI (Coef. = 0.2174, *p* = 0.024), confirming that the main findings are not sensitive to how the digital transformation process is measured. This also supports the view that DIF plays a distinct and stable role within the broader digitalization landscape.

**Table 5:** Regression using DE as the core explanatory variable.

Variables	Model(1)	Model(2)
DE	0.3705^**^	0.2174^**^
	(0.1773)	(0.0917)
FCI	—	0.017
		(0.0105)
GOV	—	–0.00188
		(0.0019)
OPEN	—	–0.1209^***^
		(0.0387)
EDU	—	–0.4925^***^
		(0.1846)
MPP	—	0.0002
		(0.0005)
Constant	0.1338^***^	0.1729^**^
	(0.0129)	(0.0361)
Year fixed effect	Yes	Yes
Provinces fixed effect	Yes	Yes
Obs	403	403
R-squared	0.7344	0.7929

Note: ^***^p < 0.01, ^**^p < 0.05,  *p* < 0.1. Robust standard error for province-level clustering in parentheses.

### Excluding direct-administered municipalities

To address potential sample heterogeneity, we exclude the four direct-administered municipalities—Beijing, Shanghai, Tianjin, and Chongqing—which differ markedly from provinces in governance structure, economic scale, and urban-rural configurations. The results (Table 6) show that DIF remains significantly and positively associated with URI (Coef. = 0.0348, *p* < 0.01), and the within-R^2^ rises to 0.9328. These findings confirm that the observed relationship is not driven by high-income outliers and holds across more homogeneous regional samples.

**Table 6:** Robustness Tests and Instrumental Variable (IV) Estimation Results

Panel A: Robustness Tests					
Variables	Excluding Municipalities	Placebo Test	Lag 1	Lag 2	Lag 3
DIF	0.0348^***^	—	0.1519^***^	0.1549^***^	0.1324^***^
	(0.0076)		(0.0468)	(0.0515)	(0.0571)
placebo_DIF	—	0.000137	—	—	—
		(0.000248)			
Control variables	Yes	Yes	Yes	Yes	Yes
Year fixed effect	Yes	Yes	Yes	Yes	Yes
Province fixed effect	Yes	Yes	Yes	Yes	Yes
R^2^	0.9328	0.9427	0.810	0.809	0.807
N	351	403	372	341	310
**Panel B: IV Diagnostics**					
			**Lag 1**	**Lag 2**	**Lag 3**
F-stat (weak ID test)	—	—	2580.87	556.90	308.41
Hansen J (*p*-value)	—	—	—	—	—

Note: ^***^p < 0.01, ^**^p < 0.05,  *p* < 0.1. Robust standard errors clustered at the province level in parentheses. Weak-ID test refers to Kleibergen–Paap rk Wald F statistic. Critical value from Stock–Yogo (2005).

### Placebo test

To test for spurious correlation, we construct a placebo variable by randomly assigning DIF values to half the sample and setting the rest to zero. The placebo regression (Table 6) yields an insignificant coefficient (Coef. = 0.000137, *p* = 0.585), well above any standard significance threshold. This suggests that the relationship between DIF and URI in the main model is unlikely to result from random chance or omitted variable bias, further validating the causal interpretation of Hypothesis 1.

### Addressing endogeneity through instrumental variables

To address potential endogeneity arising from reverse causality and omitted variables, we adopt an instrumental variable (IV) approach to reinforce the causal interpretation of the relationship between DIF and URI in the baseline fixed-effects and threshold models. Given the limited availability of strong external instruments, we adopt a two-stage least squares (2SLS) framework using lagged DIF values as internal instruments. This approach is widely accepted in macro-regional analyses where exogenous policy shocks are difficult to obtain [39].

#### Instrumental validity and relevance.

We employ the first, second, and third lags of DIF as alternative instruments and estimate the models using province-clustered robust standard errors. The first-stage regressions confirm that lagged DIF strongly predicts current DIF, with Kleibergen–Paap rk Wald F-statistics of 2,580.87, 556.90, and 308.41 for the first-, second-, and third-lag specifications, respectively—all far exceeding the Stock–Yogo critical value of 16.38 for a 10% maximal IV bias. According to the rule-of-thumb proposed by Stock and Yogo (2005) [40], F-statistics above 16.38 indicate strong instrument relevance; our values are therefore well above this benchmark. These results decisively reject the weak instrument hypothesis. The corresponding underidentification tests (Kleibergen–Paap LM $\chi^{2}$) are all highly significant (*p* < 0.001), confirming instrument relevance.

#### Exogeneity and overidentification.

Since only one excluded instrument is used in each specification, the model is exactly identified. The Hansen J-statistics therefore indicate no evidence of instrument invalidity. In the dynamic GMM framework (Arellano–Bond two-step estimation), the AR(1) and AR(2) tests show no serial correlation in the differenced residuals, and both the Sargan and Hansen tests support the joint validity of instruments (*p* = 1.000), implying that the chosen lagged DIF instruments satisfy the orthogonality condition.

#### Second-stage estimation and robustness.

The 2SLS estimates remain stable across all specifications. The coefficient of DIF on URI is 0.152 (*p* < 0.01) when using the first lag as an instrument, and 0.155 (*p* < 0.01) with the second lag, confirming the robustness of the positive and significant causal relationship. These results are consistent with the Arellano–Bond system GMM estimates, which yield a positive though less precisely estimated coefficient for DIF. The declining magnitude across higher lags (0.73 → 0.53 → 0.35 in the baseline dynamic model) suggests that the impact of digital finance on integration operates cumulatively, with stronger short- to medium-term effects as digital ecosystems mature.

Overall, the diagnostic tests provide strong evidence that lagged DIF serves as a valid and powerful instrument, mitigating weak identification and endogeneity concerns. Nonetheless, the use of internal instruments cannot fully eliminate the possibility that long-run regional attributes (e.g., institutional inertia or persistent policy bias) influence both DIF and URI. To address this, we include a comprehensive set of fiscal, institutional, and openness controls, as well as province and year fixed effects, which substantially reduce omitted variable bias. Future work could leverage quasi-natural experiments—such as phased digital finance pilot zones or exogenous expansions of mobile infrastructure—to further validate the causal link. Detailed first-stage diagnostics and instrument validity tests are presented in S7 Appendix (S7 Table), confirming the robustness of the IV identification strategy.

## Threshold effects test

### Threshold identification and statistical significance

To examine potential nonlinearities in the relationship between DIF and URI, we adopt the non-linear panel threshold regression framework of Hansen (1999)[5]. Bootstrap-based inference is applied to ensure robustness against heteroskedasticity and small-sample bias. Each threshold test employs 1,000 bootstrap replications to obtain empirical p-values and 95 percent confidence intervals for threshold parameters.

Table 7 reports the threshold test results for DIF, its sub-indices (D1-D3), and TF. The results reveal strong and statistically significant threshold effects for DIF and most sub-indices. Both single- and double-threshold models for the composite DIF index are significant at the 1 percent level (F = 445.79 and 154.23, p = 0.000), whereas the triple-threshold model is rejected (p = 0.913). Similar patterns hold for D1 and D3, while only single thresholds are detected for D2 and TF. These findings support the existence of nonlinear, regime-dependent effects in the DIF–URI nexus.

**Table 7:** Threshold Effect Tests for DIF and Sub-indices

Threshold variables	Number of Thresholds	RSS	SME	F-Value	P-Value	Threshold Value	95% Confidence Interval
DIF	Single Threshold	0.0840	0.0002	445.79^***^	0.0000	4.1133	[4.1031, 4.1292]
	Double Threshold	0.0602	0.0002	154.23^***^	0.0000	4.1292 / 2.8637	[4.1082, 4.1788] / [2.8277, 2.8914]
	Triple Threshold	0.0509	0.0001	71.51	0.913	1.2321	[1.0615, 1.2706]
D1	Single Threshold	0.0882	0.0002	405.53^***^	0.0000	4.1331	[4.0749, 4.1593]
	Double Threshold	0.0607	0.0002	176.48^***^	0.0000	4.3153 / 3.2375	[3.1765, 3.2538]
D2	Single Threshold	0.0832	0.0002	453.14^***^	0.0000	4.4379	[–]
D3	Single Threshold	0.1047	0.0003	280.58^***^	0.0000	4.4904	[4.4624, 4.5008]
	Double Threshold	0.0820	0.0002	107.90^***^	0.0001	4.4904 / 3.7948	[4.4624, 4.5008] / [3.7931, 3.7962]
TF	Single Threshold	0.1433	0.0004	99.72^**^	0.060	0.0194	[0.0029, 0.0450]

Notes: ^***^p < 0.01, ^**^p < 0.05,  *p* < 0.1. Bootstrap *p*-values and 95% confidence intervals are based on 1,000 replications following Hansen (1999). Thresholds are considered significant when F statistics exceed the 5 percent critical value.

### Economic interpretation of threshold effects

The estimated thresholds carry distinct economic implications (see Table 8). For the overall DIF index, the lower threshold (2.8637) marks the emergence of basic digital finance infrastructure such as mobile payments and online banking; the upper threshold (4.1133) signals a mature digital ecosystem featuring platform interoperability and service diversification. Below the upper threshold, the marginal effect of DIF is modest (β=0.0159, *p* < 0.01); beyond it, the coefficient rises sharply to 0.2279 (*p* < 0.01), demonstrating super-linear returns once digital maturity is achieved.

**Table 8:** Regime-specific coefficient estimates.

Regime Interval	Coefficient	Std. Err.	t-Value	P-Value	95% Confidence Interval	Number of obs
**Panel A. DIF**						
≤4.1133	0.0159^***^	0.0014	11.21	0.000	[0.0130, 0.0188]	384
>4.1133	0.2279^***^	0.0483	4.72	0.003	[0.1098, 0.3460]	19
>2.8637 (double)	0.0405^***^	0.0141	2.87	0.008	[0.0116, 0.0694]	180
**Panel B. D1**						
≤4.1133	0.0138^***^	0.0029	4.82	0.000	[0.0008, 0.0196]	388
>4.1133	0.1234*	0.0595	2.08	0.083	[–0.0221, 0.2690]	15
2nd regime	0.0602^***^	0.0152	3.95	0.000	[0.0291, 0.0914]	116
**Panel C. D2**						
≤4.4379	0.0146^***^	0.0016	8.96	0.000	[0.0112, 0.0179]	394
**Panel D. D3**						
≤4.4904	0.0084^***^	0.0012	6.83	0.000	[0.0058, 0.0109]	384
>4.4904	0.0795^***^	0.0122	6.51	0.000	[0.0514, 0.1077]	19
>3.7948 (Double)	0.0180^**^	0.0071	2.55	0.016	[0.0036, 0.0325]	184
**Panel E. TF**						
≤0.0194	0.0409^***^	0.0014	3.86	0.001	[0.0193, 0.0627]	384
>0.0194	0.5910	0.4083	1.45	0.198	[–0.4080, 1.5899]	19

Notes: ^***^p < 0.01, ^**^p < 0.05,  *p* < 0.1. Robust standard errors clustered at the province level are reported in parentheses. Regime-specific observations are reported to clarify estimation coverage under each threshold interval.

Sub-indices analyses further clarify underlying mechanisms:

- **D1** shows minimal impact below its threshold but a substantial gain after surpassing 4.1331 (β=0.1234, *p* = 0.083), reflecting scale-dependent improvements once coverage broadens.
- **D2** remains effective even below 4.4379 (β=0.0146, *p* < 0.01), underscoring the role of active digital-service usage in promoting inclusivity through reduced transaction costs.
- **D3** reveals significant nonlinearity, with coefficients jumping from 0.0084 to 0.0795 after the 4.4904 threshold (*p* < 0.01).
- For **TF**, the threshold at 0.0194 is only marginally significant (*p* = 0.060), and post-threshold effects are insignificant, suggesting that traditional finance lacks the transformative returns observed in digital domains.

### Regime-specific estimates and data constraints

Table 8 presents regime-specific coefficient estimates for DIF and its components. For DIF, effects increase tenfold beyond the upper threshold, confirming strong nonlinearities. D1 exhibits similar patterns; D2 retains low-regime significance but has insufficient upper-regime data; D3 demonstrates robust double-threshold effects with statistical significance; TF shows no meaningful post-threshold gain.

**Fig 2:**
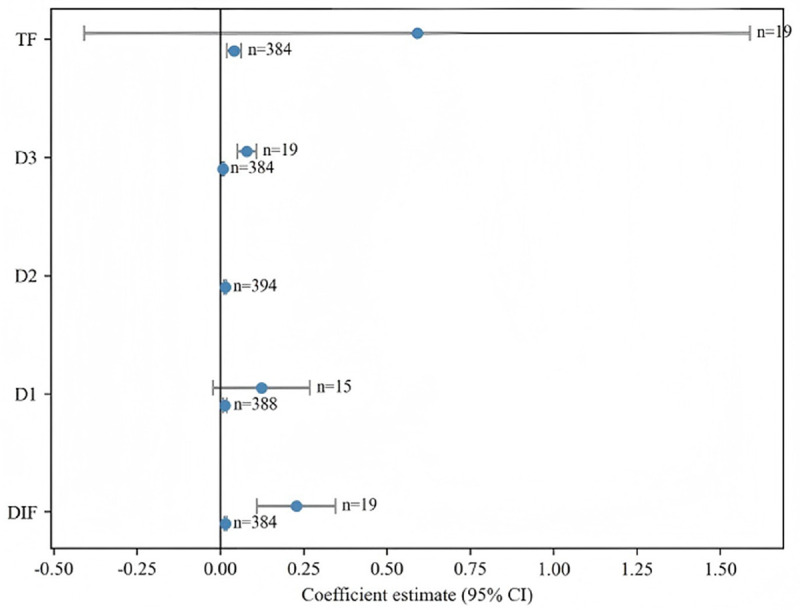
Regime-specific coefficient estimates with 95% confidence intervals. *Notes*: Each point denotes the estimated coefficient; horizontal bars show 95% confidence intervals based on province-level cluster-robust standard errors. Upper regimes (e.g., DIF >4.1133, *n* = 19) exhibit wider intervals due to limited sample size.

Table 8 reports regime-specific coefficients with robust standard errors and 95% confidence intervals. Some upper-regime estimates are based on limited observations (e.g., only *n* = 19 for DIF >4.1133). To convey this uncertainty, we (i) report 95% confidence intervals for regime coefficients in Table 8, and (ii) plot coefficient estimates with 95% confidence bands in Fig 2. The wide intervals in high-regime segments signal low precision; therefore, although point estimates often imply large economic effects, we interpret these high-threshold magnitudes cautiously.

Accordingly, these findings are interpreted conservatively: while statistical significance indicates regime-dependent effects, economic magnitudes remain imprecise and should be validated with longer time series or micro-level data. Bootstrap-based confidence intervals for threshold estimates are reported in S8 Appendix to further assess sampling variability.

### Heterogeneity, data limitations, and mechanism differentiation.

The threshold regression results reveal strong nonlinearities in the DIF–URI relationship but also highlight important heterogeneity across dimensions and regimes. While the identified thresholds are statistically robust under 1,000 bootstrap replications, the distribution of observations is highly uneven—particularly in upper-threshold intervals (e.g., DIF >4.1133, D2 >4.4379, D3 >4.4904). These sparse upper regimes account for fewer than 5% of the total sample, reflecting the early and uneven stage of digital-finance development across Chinese provinces.

Consequently, although some upper-regime coefficient estimates are statistically significant, their economic magnitudes should be interpreted with caution due to limited precision and small sample sizes.

Beyond data constraints, the heterogeneous regime behavior underscores multi-layered mechanisms through which digital finance affects URI:

- **Coverage breadth (D1)** operates via infrastructure accumulation: once sufficient reach is achieved, network externalities and scale economies emerge, amplifying integration effects.
- **Usage depth (D2)** functions through behavioral engagement: even at lower maturity levels, active participation in digital services reduces transaction costs and improves resource allocation.
- **Digitalization level (D3)** reflects technological sophistication: its high threshold (∼4.49) suggests that only advanced regions can fully leverage automation, AI-based credit scoring, and big-data analytics to enhance integration efficiency.

These differentiated pathways confirm that digital finance follows a staged, regime-dependent diffusion process rather than a linear progression. Early-stage gains stem from access expansion (D1), mid-stage gains from user adoption and service diversification (D2), and late-stage gains from technological upgrading and platform integration (D3). This pattern aligns with threshold theory, where marginal returns shift qualitatively upon crossing key enabling conditions such as institutional readiness or technological maturity.

**Fig 3:**
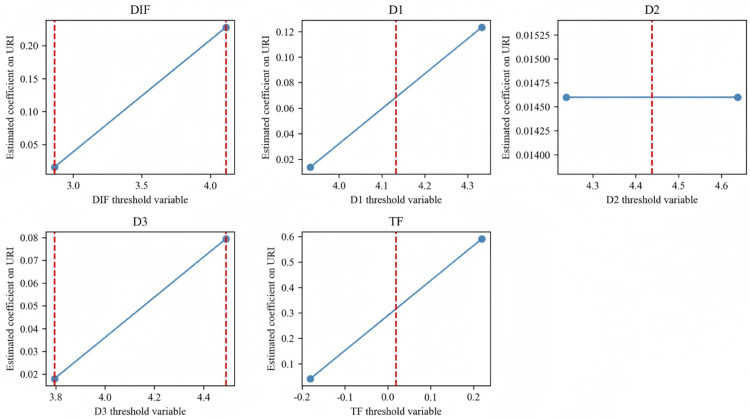
Threshold effects of DIF and sub-dimensions on URI. *Notes*: The red dashed vertical lines indicate estimated threshold values derived from Table 8. Blue lines represent estimated coefficients in lower and upper regimes; blue dots mark point estimates in each regime. The visible structural breaks reflect regime-dependent nonlinearities, reinforcing the presence of critical development inflection points in the DIF–URI relationship.

Fig 3 visualizes the threshold effects for the composite DIF index and its sub-indices. The visible jumps at threshold points corroborate the presence of critical inflection levels, confirming that digital finance exhibits super-linear yet regime-bound effects on URI.

While the results are robust to bootstrap resampling, upper-regime findings remain sensitive to data limitations. Future research should extend temporal coverage or utilize micro-level data to verify whether these super-linear effects persist in later stages of digital-finance evolution. Incorporating dynamic threshold estimation and regional spillover modeling would further enhance understanding of how digital finance interacts with local institutions, human capital, and technological ecosystems.

### Policy implications and theoretical contributions

The empirical results, reinforced by 1,000 bootstrap replications, provide robust and nuanced policy implications.

First, verified threshold effects imply that developmental returns to digital finance are regime-dependent rather than linear. Bootstrap-based inference confirms the statistical stability of these nonlinearities, suggesting that policy interventions should be phased and region-specific rather than uniform nationwide. Provinces approaching critical DIF thresholds—especially those near 2.86 and 4.11—represent high-potential targets for catalytic investment and institutional support. Prioritizing transitional regions can yield disproportionately high payoffs once digital maturity is reached.

Second, sub-dimensional findings highlight complementary but asymmetric roles of DIF components:

- **Expansion of coverage (D1)** forms the infrastructural foundation;
- **Usage depth (D2)** ensures behavioral engagement;
- **Digitalization level (D3)** drives technology-enabled efficiency gains.

However, upper-regime estimates—though statistically significant—exhibit wide confidence intervals due to limited observations, implying that their economic magnitudes should be interpreted as potentially transformative yet less precise. Policymakers should adopt an evidence-sensitive sequencing strategy, combining infrastructure investment with capability-building and digital literacy programs.

Third, the analysis clarifies that improving access alone is insufficient. Sustainable integration requires deep digital engagement and institutional embedding of digital financial systems. The identification of threshold-dependent dynamics suggests that inclusive finance policies must evolve beyond “access-first” paradigms toward **maturity-driven inclusion**, where the interplay of technology, usage, and governance determines long-term outcomes.

From a theoretical standpoint, this study enriches the literature by empirically demonstrating the threshold-dependent and regime-specific nature of digital finance’s developmental impact. These results substantiate Hansen’s (1999) threshold theory in a digital economy context, illustrating how systemic returns change qualitatively once key technological or institutional parameters surpass critical levels. The observed multi-regime behavior aligns with theories of staged technological diffusion and endogenous financial modernization.

Moreover, by integrating bootstrap-based inference with multidimensional measurement of URI, this study advances methodological rigor in identifying nonlinear policy thresholds. It contributes to bridging the gap between linear empirical models and the complex realities of digital transformation.

Future research may extend this framework by exploring:

- Dynamic threshold shifts,
- Spatial spillovers,
- Interaction effects among digital, institutional, and human-capital dimensions.

## Discussion and conclusion

This study provides new evidence on how DIF shapes URI through differentiated and regime-dependent mechanisms. The empirical patterns closely align with the theoretical channels developed in the conceptual framework while extending existing knowledge on digital-finance-driven development.

Across all specifications, usage depth (D2) consistently emerges as the strongest contributor to URI. Regular engagement with digital financial services eases liquidity constraints, strengthens household and firm-level activity, and supports more diversified rural economies. These dynamics reinforce the core proposition that digital finance enhances integration primarily by empowering users to participate more fully in economic and social life.

The digitalization dimension (D3) displays pronounced threshold effects. Its impact intensifies only once regions achieve sufficient digital maturity, indicating that data infrastructures, platform interoperability, and automated processes become truly transformative only after reaching a critical scale. This pattern highlights an institutional-transformation channel in which digital technologies reshape governance and service delivery unevenly across stages of development.

Coverage breadth (D1) plays an enabling but limited role. Expanded access facilitates entry into the digital system but cannot substitute for deep usage or institutional readiness. Effective digital-finance development therefore requires a shift from “access expansion” toward service quality, reliability, and the meaningful use of digital tools.

These findings contribute to the literature in several respects. Previous studies emphasize poverty reduction or income redistribution; by contrast, our multidimensional URI index shows that digital finance also improves spatial mobility, public-service access, and ecological coordination. The identification of explicit financial and technological thresholds helps reconcile mixed results in earlier work and provides structural explanations for why the benefits of digital finance materialize unevenly across regions.

The decomposition of openness and education effects yields a further insight. Their negative coefficients do not indicate adverse impacts but reflect spatial-concentration mechanisms, whereby gains from trade and human capital accrue disproportionately to metropolitan areas. Policymaking aimed at promoting integration must therefore focus on improving the geographic diffusion of educational and trade-related benefits rather than reducing openness itself.

A related implication concerns the emerging risks associated with highly concentrated digital-finance ecosystems. When a small number of dominant platforms shape market access and data flows, lock-in effects and opaque algorithmic practices may limit mobility and disadvantage users with low digital footprints. Ensuring transparent data governance, strengthening oversight, and broadening the diversity of digital-finance channels are important steps toward safeguarding equitable integration as digitalization accelerates.

Although the empirical analysis focuses on China, the findings carry broader implications. Many developing economies—particularly those in Asia, Latin America, and Africa—exhibit similar patterns of uneven digital infrastructure, fragmented financial access, and regionally heterogeneous development. The threshold-based framework used here offers a generalizable analytical tool for studying digital-finance-led integration in contexts where the digital divide and uneven institutional maturity constrain inclusive growth. What varies across countries is not the qualitative logic of threshold effects, but the specific levels at which thresholds occur and the speed at which digital capacity accumulates. Cross-country applications of this framework could therefore shed light on how digital finance interacts with different regulatory systems, cultural adoption patterns, and technological baselines.

Despite its contributions, the study has limitations. Although two-way fixed effects and multiple robustness checks mitigate major sources of endogeneity, reverse causality and unobserved time-varying influences cannot be fully ruled out. Future research could draw on policy shocks, quasi-natural experiments, or micro-level digital-transaction data to improve causal identification. Moreover, examining complementarities between digital finance and other structural policies—such as fiscal transfers, industrial upgrading, and environmental governance—would yield a fuller understanding of integration dynamics.

In conclusion, the capacity of digital finance to promote integration depends not only on access but also on usage depth, digital maturity, and institutional adaptability. When these conditions align and key thresholds are surpassed, DIF becomes a potent driver of balanced and resilient development. Although this study focuses on China’s provincial context, its multidimensional and threshold-based framework offers a replicable approach for analyzing digital-finance-induced integration in other developing economies undergoing uneven digital transformation.

## Supporting information

S1 File Dataset for PONE-D-25-41664: https://figshare.com/articles/dataset/data_for_PONE-D-25-41664/30656798. (PDF)

## References

[1] Zhou H, Fang CJ. The scientific connotation and internal logic of urban-rural integrated development. Finance and Trade Research. 2023;34(09):17–23.

[2] Liu Y, Zang Y, Yang Y. China’s rural revitalization and development: theory, technology and management. Journal of Geographical Sciences. 2020;30(12):1923–42. doi: 10.1007/s11442-020-1819-3

[3] Sun F. Research on the development of urban and rural development under the vision of Chinese modernization. Journal of Theory. 2023;6:152–8.

[4] Cui JJ, Zhao DY. Can digital inclusive finance promote urban-rural integration?—Empirical test based on threshold effect model. Inquiry into Economic Issues. 2023;34(09):17–23.

[5] Hansen BE. Threshold effects in non-dynamic panels: estimation, testing, and inference. Journal of Econometrics. 1999;93(2):345–68. doi: 10.1016/s0304-4076(99)00025-1

[6] King RG, Levine R. Finance, entrepreneurship and growth. Journal of Monetary Economics. 1993;32(3):513–42. doi: 10.1016/0304-3932(93)90028-e

[7] McKinnon RI. The value-added tax and the liberalization of foreign trade in developing economies: a comment. Journal of Economic Literature. 1973;11:520–4.

[8] Jeanneney SG, Kpodar K. Financial development and poverty reduction: can there be a benefit without a cost?. Journal of Development Studies. 2011;47(1):143–63. doi: 10.1080/00220388.2010.506918

[9] Seven U, Coskun Y. Does financial development reduce income inequality and poverty? Evidence from emerging countries. Emerging Markets Review. 2016;26:34–63. doi: 10.1016/j.ememar.2016.02.002

[10] Park D, Shin K. Economic growth, financial development, and income inequality. Emerging Markets Finance and Trade. 2017;53:2794–825.

[11] Peng H, Wang J, Wen L, Ding P, Zhu Y. Is the development of inclusive finance truly able to alleviate poverty?—An empirical study based on spatial effect and threshold effect. Emerging Markets Finance and Trade. 2021;58(9):2505–21. doi: 10.1080/1540496x.2021.2002141

[12] Demirguc-Kunt A, Klapper L. Measuring financial inclusion: the global findex database. WPS 6025 . Washington, DC: World Bank; 2012. http://documents.worldbank.org/curated/en/453121468331738740

[13] Mao F, Wang Y, Zhu M. Digital financial inclusion, traditional finance system and household entrepreneurship. Pacific-Basin Finance Journal. 2023;80:102076. doi: 10.1016/j.pacfin.2023.102076

[14] Shen Y, Jing Y, Liu Y. Unveiling the spatial coupling dynamics and coordination mechanisms between digital inclusive finance and rural industrial integration development. Land. 2025;14(3):499. doi: 10.3390/land14030499

[15] Marshall A, Dezuanni M, Burgess J, Thomas J, Wilson CK. Australian farmers left behind in the digital economy – insights from the Australian digital inclusion index. Journal of Rural Studies. 2020;80:195–210. doi: 10.1016/j.jrurstud.2020.09.001

[16] Thomä J. An urban-rural divide (or not?): small firm location and the use of digital technologies. Journal of Rural Studies. 2023;97:214–23. doi: 10.1016/j.jrurstud.2022.12.020

[17] Ran M, Chen L, Li W. Financial deepening, spatial spillover, and urban–rural income disparity: evidence from China. Sustainability. 2020;12(4):1450. doi: 10.3390/su12041450

[18] Ge H, Li B, Tang D, Xu H, Boamah V. Research on digital inclusive finance promoting the integration of rural three-industry. International Journal of Environmental Research and Public Health. 2022;19(6):3363. doi: 10.3390/ijerph19063363 35329048 PMC8954514

[19] Li W, Zhang L, Pu M, Wang H. Digital inclusive finance, rural revitalization and rural consumption. PLoS One. 2025;20(1):e0310064. doi: 10.1371/journal.pone.0310064 39752429 PMC11698437

[20] Chakravarty SR, Pal R. Financial inclusion in India: an axiomatic approach. Journal of Policy Modeling. 2013;35(5):813–37. doi: 10.1016/j.jpolmod.2012.12.007

[21] Frost J, Gambacorta L, Gambacorta R. On the nexus between wealth inequality, financial development and financial technology. Journal of Economic Behavior & Organization. 2022;202:429–51. doi: 10.1016/j.jebo.2022.08.011

[22] Gallego-Losada M-J, Montero-Navarro A, García-Abajo E, Gallego-Losada R. Digital financial inclusion. Visualizing the academic literature. Research in International Business and Finance. 2023;64:101862. doi: 10.1016/j.ribaf.2022.101862

[23] Lian X, Mu Y, Zhang W. Digital inclusive financial services and rural income: evidence from China’s major grain-producing regions. Finance Research Letters. 2023;53:103622. doi: 10.1016/j.frl.2022.103622

[24] Upadhyay P, Kumar A, Dwivedi YK, Adlakha A. Continual usage intention of platform-based governance services: a study from an emerging economy. Government Information Quarterly. 2022;39(1):101651. doi: 10.1016/j.giq.2021.101651

[25] Papaskiri TV, Kasyanov AE, Alekseenko NN, Semochkin VN, Ananicheva EP, Volkov IV. Modern technologies of digital land management. IOP Conf Ser: Earth Environ Sci. 2019;350(1):012066. doi: 10.1088/1755-1315/350/1/012066

[26] Magomedov IA, Murzaev HA, Bagov AM. The role of digital technologies in economic development. IOP Conference Series: Materials Science and Engineering. 2020;862(5):052071. doi: 10.1088/1757-899x/862/5/052071

[27] Liu J, Puah C-H, Arip MA, Jong M-C. Impacts of digital financial inclusion on urban–rural income disparity: a comparative research of the Eastern and Western Regions in China. Economies. 2023;11(11):282. doi: 10.3390/economies11110282

[28] Wu Z, Qiu W, Wang L. Digital economy and urban and rural integration, development research. FHSS. 2023;3(11):49–59. doi: 10.54691/fhss.v3i11.5744

[29] Hao Y, Zhang B, Yin H. Can digital finance drive urban–rural integration?. Economic Research-Ekonomska Istraživanja. 2023;36(2). doi: 10.1080/1331677x.2023.2169736

[30] Schmied J, Marr A. Financial inclusion and poverty: the case of Peru. Regional and Sectoral Economic Studies. 2016;16(2):29–43.

[31] Dai F, Liu H, Zhang X, Li Q. Does the equalization of public services effect regional disparities in the ratio of investment to consumption? Evidence from provincial level in China. Sage Open. 2022;12(1). doi: 10.1177/21582440221085007

[32] Zhang X, Zhang J, Wan G, Luo Z. Fintech, growth and inequality: evidence from China’s household survey data. Singapore Economic Review. 2020;65(supp01):75–93. doi: 10.1142/s0217590819440028

[33] Zhou JN, Zou W, Qin FC. Review of urban-rural multi-dimensional integration and influencing factors in China based on the concept of equivalence. Geographical Research. 2020;39(8):1836–51. doi: 10.11821/dlyj020190572

[34] Xin-lin Z, Fang-dao Q, Chuan-geng Z. Evolution of urban-rural integration in Huaihai Economic Zone from the perspective of spatio-temporal interaction. Journal of Natural Resources. 2020;35(8):1867. doi: 10.31497/zrzyxb.20200809

[35] Wang Y, Zhang Z, Xu SY, Liu R, Zhu YM. Does digital inclusive finance really promote urban-rural integration? Empirical verifications based on China’s inter-provincial panel data. Journal of Nanjing University of Science and Technology (Social Sciences). 2023;36(5):37–50. doi: 10.19847/j.ISSN1008-2646.2023.05.006

[36] Guo F, Wang J, Wang F, Kong T, Zhang X, Cheng Z. Measuring the development of digital inclusive finance in China: index compilation and spatial characteristics. Quarterly Journal of Economics. 2020;19(04):1401–18. doi: 10.13821/j.cnki.ceq.2020.03.12

[37] Bukht R, Heeks R. Digital economy policy in developing countries. Development Informatics Group, University of Manchester; 2018. 10.13140/RG.2.2.24272.15364

[38] Ozil PK. Digital finance research and developments around the world: a literature review. International Journal of Business Forecasting and Marketing Intelligence. 2023;8(1):35–51.

[39] Arellano M, Bond S. Some tests of specification for panel data: Monte Carlo evidence and an application to employment equations. The Review of Economic Studies. 1991;58(2):277. doi: 10.2307/2297968

[40] Stock J, Yogo M. Testing for weak instruments in linear IV regression. Identification and inference for econometric models. New York: Cambridge University Press; 2005. p. 80–108.

