# Supplementary material for: Unveiling nonlinear effects of Digital Inclusive Finance on urban-rural integration: A threshold panel analysis of China
Source: PLoS One. 2026 Feb 20;21(2):e0342432. doi: 10.1371/journal.pone.0342432 (PMC12923127; doi:10.1371/journal.pone.0342432)
Supplement: S1 File — (PDF) [file pone.0342432.s001.pdf]

Supporting Information

S1 File. Dataset for PONE-D-25-41664:
`https://figshare.com/articles/dataset/data_for_PONE-D-25-41664/30656798`

S2 Table1. Indicator System for Urban-Rural Integration (URI)

Dimension	Indicator	Attribute	Weight
Economic Dimension	Urban-Rural Per Capita Income Ratio (%)	Negative	0.008739
	Urban-Rural Per Capita Consumption Expenditure Ratio (%)	Negative	0.000444
	Urban-Rural Per Capita Wage Income Ratio (%)	Negative	0.003940
	Urban-Rural Engel Coefficient Ratio (%)	Positive	0.001152
	Dual Economy Contrast Index (%)	Positive	0.025840
	Per Capita GDP	Positive	0.033309
	Non-agricultural to Agricultural Output Ratio (%)	Positive	0.219681
	Agricultural Fiscal Expenditure Ratio (%)	Positive	0.018796
	Industrial Output Comparison (%)	Negative	0.006532
	Industrial Synchronization Index (%)	Negative	0.007906
Social Dimension	Urban-Rural Education and Entertainment Expenditure Ratio (%)	Negative	0.000801
	Urban-Rural Transportation and Communication Expenditure Ratio (%)	Negative	0.006179
	Urban-rural ratio of physicians per 1,000 population	Negative	0.001036
	Urban-Rural Per Capita Healthcare Expenditure Ratio (%)	Negative	0.005055
	Urban-Rural Income and Expenditure Structure Disparity (%)	Negative	0.000413
	Urban-Rural Pension Insurance Coverage Ratio (%)	Positive	0.030414
	Urban-Rural Medical Security	Positive	0.016748
Spatial Dimension	Spatial Mobility Medium	Positive	0.027715
	Urban-Rural Land Allocation (%)	Positive	0.137860
	Internet Penetration Rate	Positive	0.030409
	Transportation Network Density	Positive	0.032387
	Urban-Rural Spatial Circulation Entity	Positive	0.048463
	Urbanization Level of Land Use (%)	Positive	0.020302
Ecological Dimension	Electricity Consumption per Unit of GDP	Negative	0.012721
	Industrial Waste Gas Emissions	Negative	0.007716
	Environmental Protection Expenditure (%)	Positive	0.020827
	Forest Coverage Rate (%)	Positive	0.035548
	Pollution Control Investment Ratio(%)	Positive	0.065297
Population Dimension	Non-agricultural to Agricultural Employment Ratio(%)	Positive	0.152820
	Urbanization Rate of Population(%)	Positive	0.010476
	Urban-Rural Population Structure(%)	Negative	0.010476

Note: All weights are derived from the entropy weight method.

S2 Appendix

S2 Table2. Variable Definitions for Regression Analysis

Variable	Definition	Unit	Source
URI	Urban-Rural Integration Index (composite index of 31 indicators)	Index (0–1)	Calculated by authors using data from China Statistical Yearbook and provincial statistics
DIF	Digital Inclusive Finance Index	Index (rescaled by dividing by 100)	Peking University Digital Finance Research Center (`https://idf.pku.edu.cn`)
Breadth (D1)	Coverage breadth of digital finance	Index	Same as above
Depth (D2)	Usage depth of digital finance	Index	Same as above
Depth (D3)	Level of digital service provision	Index	Same as above
FCI	Level of material capital investment (ratio of total fixed asset investment to GDP)	%	China Statistical Yearbook
OPEN	Extent of economic openness (ratio of total import and export trade to GDP)	%	Same as above
EDU	Level of education development (ratio of fiscal education expenditure to GDP)	%	Same as above
GOV	Level of financial support for rural and agricultural development	%	Same as above
MPP	Level of mobile phone adoption (number of mobile phones per 100 people)	—	Same as above
TF	Traditional Financial Development Index	Index	China Financial Yearbook; the China Banking and Insurance Statistical Bulletin

S3 Appendix: Entropy Weight Method for Composite Index Construction

To objectively determine indicator weights for the Urban–Rural Integration (URI) Index, we apply the entropy weight method. The steps are as follows:

Step 1: Data Standardization

For positive indicators:

$$x'_{ij} = \frac{x_{ij} - \min(x_j)}{\max(x_j) - \min(x_j)}$$

For negative indicators:

$$x'_{ij} = \frac{\max(x_j) - x_{ij}}{\max(x_j) - \min(x_j)}$$

where x_{ij} is the original value of indicator j for region i, and x'_{ij} is the standardized value.

Step 2: Proportion Calculation

$$p_{ij} = \frac{x'_{ij}}{\sum_{i=1}^{n} x'_{ij}}$$

Step 3: Entropy Calculation

$$e_j = -k \sum_{i=1}^{n} p_{ij} \ln(p_{ij}), \quad k = \frac{1}{\ln(n)}$$

Step 4: Redundancy and Weight Assignment

$$w_j = \frac{1 - e_j}{\sum_{j=1}^{m}(1 - e_j)}$$

where w_j is the final weight assigned to indicator j, and m is the number of indicators.

Step 5: Composite Index Aggregation

$$\text{URI}_i = \sum_{j=1}^{m} w_j x'_{ij}$$

Rationale: The entropy weight method assigns higher weights to indicators with greater variability, reflecting their higher information contribution to the composite index.

S4 Table. Ridge Regression Results

Variable	Ridge Coefficient	Interpretation
Financial_Index_PCA	0.006	Positive and stable effect on URI
GOV	0.0072	Small positive, not statistically critical
OPEN	0.004	Minor positive effect after penalization
EDU	-0.00007	Negligible, consistent with OLS direction
FCI	-0.0083	Slightly negative, unchanged sign
MPP	-0.0029	Similar direction as OLS
Constant	-0.0013	Statistically insignificant
Optimal λ	79.25	Selected by cross-validation

Notes: Ridge regression mitigates variance inflation by penalizing large coefficient magnitudes. All ridge coefficients maintain the same direction as those in the OLS and fixed-effects models, indicating no distortion due to multicollinearity. Standardized variables are used for comparison consistency.

S4 Table.

S5 Table1-4.

S5 Table1. Heterogeneity by Development Stage

**Variables**	**(1)** **High GDP_percapita Group**	**(2)** **Low GDP_percapita Group**	**(3)** **Interaction (GDP_percapita×DIF)**	**(4)** **Interaction (GDP×D)**
DIF	0.1095**	0.0296**	0.083***	—
	(0.0389)	(0.0119)	(0.026)	
D1	0.015	0.031**	—	0.024
	(0.057)	(0.011)		(0.019)
D2	0.067***	0.014	—	0.048***
	(0.021)	(0.008)		(0.015)
D3	0.036**	0.001	—	0.028***
	(0.015)	(0.004)		(0.010)
Control variables	Yes	Yes	Yes	Yes
Year fixed effect	Yes	Yes	Yes	Yes
Province fixed effect	Yes	Yes	Yes	Yes

Note: ***$p < 0.01$, **$p < 0.05$, *$p < 0.1$. Robust standard errors clustered at the province level are reported in parentheses.

S5 Table2. Heterogeneity by ICT Infrastructure

**Variables**	**(1)** High ICT Group	**(2)** Low ICT Group	**(3)** Interaction (ICT×DIF)	**(4)** Interaction (ICT×D)
DIF	0.112***	0.070**	0.090***	—
	(0.034)	(0.028)	(0.028)	
D1	0.020	0.019	—	0.020
	(0.038)	(0.015)		(0.018)
D2	0.057***	0.038*	—	0.053***
	(0.014)	(0.021)		(0.016)
D3	0.032**	0.029**	—	0.029***
	(0.011)	(0.012)		(0.009)
Control variables	Yes	Yes	Yes	Yes
Year fixed effect	Yes	Yes	Yes	Yes
Province fixed effect	Yes	Yes	Yes	Yes

Note: ***$p < 0.01$, **$p < 0.05$, *$p < 0.1$. Robust standard errors clustered at the province level in parentheses.

S5 Table3. Heterogeneity by Industrial Structure

**Variables**	**(1)** High Service Share	**(2)** Low Service Share	**(3)** Interaction (Service×DIF)	**(4)** Interaction (Service×D)
DIF	0.122**	0.026**	0.087***	—
	(0.041)	(0.011)	(0.027)	
D1	0.012	0.028*	—	0.023
	(0.038)	(0.015)		(0.020)
D2	0.083***	0.012*	—	0.050***
	(0.021)	(0.007)		(0.015)
D3	0.041***	0.005	—	0.029***
	(0.012)	(0.004)		(0.009)
Control variables	Yes	Yes	Yes	Yes
Year fixed effect	Yes	Yes	Yes	Yes
Province fixed effect	Yes	Yes	Yes	Yes

Note: ***$p < 0.01$, **$p < 0.05$, *$p < 0.1$. Robust standard errors clustered at the province level in parentheses.

S5 Table4. Heterogeneity by Fiscal Supports

**Variables**	**(1)** High Fiscal Support	**(2)** Low Fiscal Support	**(3)** Interaction (Service×DIF)	**(4)** Interaction (Fiscal Support×D)
DIF	0.040**	0.122**	0.086***	—
	(0.015)	(0.044)	(0.029)	
D1	0.037**	0.005	—	0.020
	(0.016)	(0.050)		(0.020)
D2	0.016*	0.084***	—	0.050***
	(0.009)	(0.023)		(0.017)
D3	0.001	0.047**	—	0.028***
	(0.005)	(0.018)		(0.010)
Control variables	Yes	Yes	Yes	Yes
Year fixed effect	Yes	Yes	Yes	Yes
Province fixed effect	Yes	Yes	Yes	Yes

Note: ***$p < 0.01$, **$p < 0.05$, *$p < 0.1$. Robust standard errors clustered at the province level in parentheses.

S6 Table. Decomposition Results

Variables	EDU split	EDU proxy	EDU interact	OPEN*Urban
DIF	0.1124***	0.1124***	0.0934***	0.0922***
	(0.0300)	(0.0300)	(0.0301)	(0.0235)
OPEN	-0.1203***	-0.1203***	-0.1337***	0.2309***
	(0.0283)	(0.0283)	(0.0186)	(0.0715)
edu_urban_proxy / edu_x_urban	-0.000014	0.000048	—	—
	(0.000021)	(0.000069)		
edu_rural_proxy	-0.000063	—	—	—
	(0.000052)			
export_share	—	—	0.0530	—
			(0.1127)	
OPEN_x_urban	—	—	—	-0.4435***
				(0.0692)
Observations	403	403	403	403
R^2	0.812	0.812	0.802	0.835

Notes: Robust standard errors in parentheses. ***$p < 0.01$, **$p < 0.05$, *$p < 0.1$. All models include province fixed effects and year dummies. The decomposition and interaction results confirm that negative baseline signs for OPEN and EDU are driven by urban-concentration effects rather than genuine disbenefits.

S7 Appendix: First-Stage Diagnostics and Instrument Validity Tests To ensure the robustness of instrumental variable estimation, S7 Table reports the full set of first-stage diagnostics from the `ivreg2` procedure, including the Kleibergen–Paap LM underidentification test, the Wald F-statistic for weak identification, and the Cragg–Donald F-statistic where applicable. All results indicate that the lagged DIF instruments are highly correlated with the endogenous variable and satisfy the relevance condition. All IV estimations were implemented in Stata 18.0 using the `ivreg2` command with province-level clustered robust standard errors.

S7 Table. First-stage Results of Instrumental Variable Estimation

Specification	Instrument	Kleibergen-Paap LM χ^2 (p-value)	Kleibergen-Paap F	Cragg-Donald F	Stock–Yogo Critical Value (10%)	Under-ID Test	Weak-ID Test
Lag1 (IV: L.DIF)	L.DIF	31.256 (0.000)	2580.87	2580.87	16.38	Reject	Strong
Lag2 (IV: L2.DIF)	L2.DIF	28.522 (0.000)	556.90	556.90	16.38	Reject	Strong
Lag3 (IV: L3.DIF)	L3.DIF	24.947 (0.000)	308.41	308.41	16.38	Reject	Strong

Notes: Reported statistics are based on ivreg2 URI (DIF = L.DIF) GOV OPEN EDU FCI MPP i.year, cluster(province_num) first and corresponding lag specifications. All p-values are robust to heteroskedasticity and clustered by province.

S8 Appendix: Visualizing Regime Uncertainty To enhance transparency and reproducibility, this appendix explains the procedure used to generate Fig.**??**, which visualizes the coefficient estimates and their 95% confidence intervals for each threshold regime.

For each estimated regime, the point estimate and its 95% confidence interval (CI) were calculated as:

$$CI_r = \hat{\beta}_r \pm 1.96 \times SE_{r,\text{clustered}}$$

where $SE_{r,\text{clustered}}$ denotes the province-level cluster–robust standard error reported in S5 Appendix Table D. These values were plotted as points (point estimates) and horizontal bars (95% confidence intervals). Regimes with small sample sizes ($n < 30$)—particularly the upper-threshold regime (e.g., DIF >4.1133, $n = 19$)—were explicitly flagged in the figure to highlight their higher sampling uncertainty.

To further assess the stability of the estimated thresholds, a bootstrap sensitivity analysis with 1,000 replications was performed. Each replication re-estimated the threshold location and regime-specific coefficients, thereby approximating the sampling distribution of estimates. The bootstrapped confidence intervals closely align with the asymptotic ones reported in Table **??**, confirming the robustness of the results and the limited bias due to small sample regimes.

Stata replication template:

```
* Bootstrap threshold estimation and store regime coefficients
bootstrap beta=_b[DIF], reps(1000) cluster(province): \\
   threg URI DIF controls, threshold(DIF)
* Compute 95% confidence intervals
gen ub = beta + 1.96*se
gen lb = beta - 1.96*se
* Plot regime-specific coefficients and confidence bands
twoway (rarea ub lb regime, color(gs14)) \\
   (scatter beta regime, msymbol(o) mcolor(black)), \\
   title("Regime-specific Estimates with 95% CIs") \\
   xtitle("Threshold Regime") ytitle("Coefficient")
```

The plotted results (see Fig.**??**) transparently convey uncertainty across threshold regimes. Wide confidence intervals in the upper regime reflect small-n estimation and thus warrant cautious interpretation. Bootstrapped confidence intervals for threshold locations and coefficients are available upon request for replication purposes.
